# Endothelial Cell Autoantibodies in Predicting Declining Renal Function, End-Stage Renal Disease, or Death in Adult Type 2 Diabetic Nephropathy

**DOI:** 10.3389/fendo.2014.00128

**Published:** 2014-08-11

**Authors:** Mark B. Zimering, Jane H. Zhang, Peter D. Guarino, Nicholas Emanuele, Peter A. McCullough, Linda F. Fried, Investigators for the VA NEPHRON-D

**Affiliations:** ^1^Medical Service, Department of Veterans Affairs New Jersey Health Care System, East Orange, NJ, USA; ^2^Rutgers-Robert Wood Johnson Medical School, New Brunswick, NJ, USA; ^3^West Haven Cooperative Studies Program Coordinating Center, Connecticut Veterans Healthcare System, West Haven, CT, USA; ^4^Veterans Affairs Hospital, Hines, IL, USA; ^5^Baylor University Medical Center, Baylor Heart and Vascular Institute, Baylor Jack and Jane Hamilton Heart and Vascular Hospital, Dallas, TX, USA; ^6^The Heart Hospital, Plano, TX, USA; ^7^Veterans Affairs Medical Center, Pittsburgh, PA, USA

**Keywords:** endothelium, autoantibodies, type 2 diabetes, nephropathy, chronic kidney disease

## Abstract

Albuminuria is a strong predictor of diabetic nephropathy chronic kidney disease outcomes. Yet, therapeutic albuminuria-lowering has not consistently translated into a reduction in clinical events suggesting the involvement of additional pathogenic factors. Our hypothesis is that anti-endothelial cell autoantibodies play a role in development and progression in diabetic nephropathy. We determined anti-endothelial cell antibody (AECA) bioactivity in protein A-elutes of baseline plasma in 305 participants in the VA NEPHRON-D study, a randomized trial of angiotensin receptor blocker (ARB) or dual ARB plus angiotensin-converting enzyme inhibitor therapy in type 2 diabetes with proteinuric nephropathy. Thirty-eight percent (117/305) of participants had significantly reduced endothelial cell survival ( ≤80%) in the IgG fraction of plasma. A VA NEPHRON-D primary endpoint [end-stage renal disease (ESRD), significant reduction in estimated glomerular filtration rate, or death] was experienced by 58 individuals. In adjusted Cox regression analysis, there was a significant interaction effect of baseline anti-endothelial cell-mediated cell survival and albuminuria on the hazard rate (HR) for primary composite endpoint (*P* = 0.017). Participants lacking strongly inhibitory antibodies with albuminuria ≥1 g/g creatinine had a significantly increased primary event hazard ratio, 3.41 – 95% confidence intervals (CI 1.84–6.33; *P* < 0.001) compared to those lacking strongly inhibitory antibodies with lower baseline albuminuria ( <1 g/g creatinine). These results suggest that anti-endothelial cell antibodies interact significantly with albuminuria in predicting the composite endpoint of death, ESRD, or substantial decline in renal function in older, adult type 2 diabetic nephropathy.

## Introduction

Diabetic nephropathy is a leading cause of end-stage renal disease (ESRD) and cardiovascular or all-cause mortality in adults worldwide (1). Renin–angiotensin system (RAS) activation plays a central role in the pathogenesis of diabetic nephropathy (2). Blockade of the RAS with either an angiotensin-converting enzyme inhibitor (ACEi) or angiotensin receptor blocker (ARB) lowers urinary albumin excretion and provides renal protection in diabetic nephropathy (2–5). Since dual blockage of the RAS results in greater albuminuria-lowering than from ACEi or ARB monotherapy alone (6), the expectation was that albuminuria-lowering *per se* may be a goal of treatment in diabetic nephropathy (7). The lack of demonstrable benefit, however, from dual (ACEi + ARB) therapy compared to mono-therapy on composite renal or cardiovascular outcomes in the Ongoing Telmisartan Alone and in Combination with Ramipril Global Endpoint Trial (ONTARGET) (8) or in the VA NEPHRON-D study (9) has refocused attention on the need to identify additional pathogenic factors (besides albuminuria) that may contribute to clinically important end point occurrences in diabetic nephropathy.

Endothelial damage and inflammation are among several non-traditional cardiovascular risk factors implicated in mediating residual risk in high-risk diabetes mellitus (DM) (10). Increased retinal and glomerular capillary permeability underlies diabetic macular edema and albuminuria and vascular permeability is a hallmark of generalized diabetic vasculopathy (11). In our prior work, strongly inhibitory endothelial cell (EC) IgG autoantibody activity was associated with the development of nephropathy in older adult type 2 DM participants in the Veterans Affairs Diabetes Trial (VADT) (12). Since anti-EC antibodies (AECA) in type 2 DM caused strong EC contraction (via Rho A/ROCK signaling pathway) or apoptosis *in vitro* (12), we hypothesized that AECA may contribute (and/or be a marker for) progression of diabetic nephropathy. In the current study, we tested whether AECA is associated with an increased risk of progression of kidney disease in the VA NEPHRON-D study (9), a randomized study comparing ARB or dual ARB plus ACEi therapy in type 2 DM having proteinuric diabetic nephropathy.

## Subjects and Methods

### Study population

The study population for VA NEPHRON-D has been previously described. In brief, participants were individuals with type 2 diabetes, urine albuminuria ≥300 mg/g creatinine, and an estimated glomerular filtration (eGFR) rate 30–89.9 ml/min/1.73 m^2^ (13). Individuals provided informed consent for biorepository samples at randomization. For the current analysis, a randomly selected subset of 305 individuals from the 1448 randomized who had biorepository samples was chosen. The primary endpoint was defined as ESRD, significant reduction in eGFR, or death. Significant reduction in estimated GFR was defined as a decline of ≥30 ml/min/1.73 m^2^ if the initial eGFR was ≥60 ml/min/1.73 m^2^ or a decline of ≥50% if the initial estimated GFR was <60 ml/min/1.73 m^2^.

Overnight fast blood draws were performed at each site. EDTA plasma was aliquoted and shipped frozen to a central repository and stored at −80°C. Archived, coded frozen EDTA plasma was shipped to the laboratory of Dr. Zimering (VA New Jersey Health Care System, Lyons, NJ, USA) where EC bioassays were performed. The samples were kept frozen (−40°C) prior to assay for protein A-eluted EC bioactivity. EC bioactivity in the protein A eluates of plasma was previously shown to be stable for 5 years or longer at −20°C (14).

### Laboratory and clinical measures

Comorbidity was determined using chart review and patient history; race and ethnicity were by self-report. Cardiovascular disease is defined as the prior occurrence of any one of the following at the baseline study evaluation visit (myocardial infarction, coronary revascularization, amputation, peripheral vascular disease, congestive heart failure, cerebrovascular event). Standard laboratory and clinical measures were determined at individual study sites. Creatinine value for the primary endpoint was measured in a central lab using an IDMS traceable creatinine assay. Estimated GFR was calculated using the Modification of Diet in Renal Disease (MDRD) formula for IDMS traceable creatinine. Urinary albumin excretion rate was determined in a random sample in the vast majority of participants.

### Endothelial cell autoantibodies

#### Protein A affinity chromatography

Protein A chromatography was carried out as previously described (14, 15). Four-tenths milliliter aliquots of plasma were adjusted to pH 8.0 by adding 0.8 mL 100 mmol/L Tris (pH 8). After syringe filtration to clarify samples, 1 mL was applied to a 1 mL column of packed protein A beads (Pierce Chemical Co., Rockford, IL, USA) equilibrated in 100 mmol/L Tris, pH 8.0. The column was washed and eluted as previously described (15). The eluate fractions containing nearly all the recovered protein were pH neutralized and stored at 0–4°C. Inhibitory activity in protein A eluate fractions was unchanged, appearing in the retentate fraction after dialysis (10 mmol/L phosphate, pH 7.4) and ultrafiltration on a 10 kDa cutoff membrane (Centricon-10; Millipore Corp., Bedford, MA, USA). All fractions were sterile filtered (Millipore Corp. Bedford, MA, USA; 0.2 um) before assay for growth-promoting activity.

#### Protein determinations

Protein concentrations were determined by a bicinchoninic acid protein assay kit (Pierce Chemical Co., Rockford, IL, USA).

#### Cell culture and growth assays

Bovine pulmonary artery (BPA) ECs (Clonetics, Inc. San Diego, CA, USA) were maintained at 37°C in 5% CO2/95% air in Medium 199 plus 10% fetal calf serum. BPA cells were passaged continuously and used between passages 4 and 10. BPA EC (M199, 10% FCS) were incubated (at 37°C× 48 h) with (30 μg/mL) of the protein A eluate of plasma. BPA ECs mitogenicity is exquisitely sensitive to low concentrations of basic fibroblast growth factor (bFGF) (16). We chose BPA ECs in order to permit sensitive detection of a dynamic range of stimulatory of inhibitory EC activity in the protein A eluates of plasma. In our prior studies, BPA ECs proliferated in response to high MW, FGF-like substances in diabetic micro- or albuminuric plasma and/or urine (17) and could be inhibited or stimulated by the protein A eluates from certain cancer sera (14).

#### Colorimetric estimation of cell number

Endothelial cell number assays were carried out as previously reported (15). Confluent cells were trypsinized and plated at 1 to 10 × 10^3^ cells/well in Medium 199 plus 10% fetal calf serum in 96-well plates. After 1 or 2 days incubation for cells to reach 60–80% confluency, test fractions (30 μg/mL of protein A eluates of plasma) were added to wells in quadruplicate. After 48 h incubation in the presence of test fractions, cells were washed with PBS and processed for the colorimetric estimation of cell number, i.e., cell-associated acid phosphatase activity, as previously described (14). There was a linear relationship between EC number and optical density at 410 nm as previously described (14). Growth-promoting activity is expressed as a percentage of the control cell number for cells grown in the absence of test protein A-eluate fractions. Antibody-mediated cell survival was defined as the proportion of surviving EC compared to cells without test IgG (100%). Strongly inhibitory antibody activity (i.e., ≤80% EC survival) corresponds to anti-EC antibody-mediated survival previously observed to correlate with the subsequent risk for progression to diabetic nephropathy (12). Each point represents the mean of quadruplicate determinations. The intra- and inter-assay coefficients of variation were 4 and 6% at 1:50 dilution of protein A-eluted fractions from plasma of three diabetic patients (*n* = 2–4 assays in each patient).

### Statistical analysis

Student’s *t*-test or Chi-square test was used for the comparisons between antibody groups shown in Table 1. Cox regression was used to model possible baseline risks factors associated with time to first post-randomization occurrence of the primary composite endpoint. Both treatment group (losartan monotherapy or losartan plus lisinopril), and the stratification variables (baseline eGFR <60 vs. ≥60), baseline albuminuria ( <1 vs. ≥1 g/g creatinine) were included as covariates in the model when testing for an effect of AECA-mediated cell survival ( ≤80% vs. >80%). In univariate regression analysis, other risk variables [age, HbA1c, systolic or diastolic blood pressure, low density lipoprotein (LDL) cholesterol concentration, and BMI] were tested for association with time to primary endpoint, and were included in the model if it reached 0.05 of significance level. Two way-interactions were checked among treatment, albuminuria, AECA-mediated cell survival, eGFR, age, and prior macrovascular disease one by one due to a limited number of events.

**Table 1:** **Participant characteristics at baseline by anti-endothelial cell antibody-mediated cell survival group**.

	AECA-mediated cell survival (≤80%)*			
	Overall (*n* = 305)	Yes (*n* = 117)	No (*n* = 188)	*P*-value
Age (year)	65.3 ± 8.4	66.3 ± 8.8	64.6 ± 8.0	0.09
Hispanic (%)	24 (7.9)	10 (8.5)	14 (7.4)	0.73
Non-Hispanic White (%)	205 (67.2)	81 (69.2)	124 (66.0)	0.55
African American (%)	70 (23.0)	23 (19.7)	47 (25.0)	0.28
BMI (kg/m^2^)	34.6 ± 6.9	34.6 ± 7.0	34.7 ± 6.8	0.94
Systolic BP (mm Hg)	136.9 ± 15.3	137.4 ± 14.1	136.7 ± 16.0	0.69
Diastolic BP (mm Hg)	73.0 ± 10.2	73.5 ± 10.8	72.7 ± 9.7	0.55
Creatinine, mg/dl geometric mean (95% CI)	1.4 (1.4–1.5)	1.4 (1.3–1.5)	1.4 (1.4–1.5)	0.97
eGFR, ml/min/1.73m^2^	55.2 ± 18.8	55.1 ± 18.5	55.2 ± 19.0	0.97
Urine albumin/creatinine,/g, median (95% CI)	1501 (466, 1824)	1579(420, 2220)	1452(481, 1696)	0.51
HbA_1_c, %	7.8 ± 1.2	7.8 ± 1.3	7.8 ± 1.2	0.95
LDL cholesterol (mg/dl)	82.7 ± 33.7	83.0 ± 33.7	82.5 ± 33.8	0.91
Current smoker (%)	65 (21.3)	22 (18.8)	43 (22.9)	0.40
H/O macrovascular event (%)	184 (60.3)	62 (53.0)	122 (64.9)	0.04
Diabetic retinopathy (%)	137 (44.9)	62 (53.0)	75 (39.9)	0.03
Peripheral neuropathy (%)	166 (54.4)	106 (56.4)	166 (54.4)	0.38
Autonomic neuropathy (%)	12 (3.9)	9 (4.8)	12 (3.9)	0.33
Atrial fibrillation (%)	28 (9.2)	15 (12.8)	13 (6.9)	0.08
Use of insulin (%)	210 (68.8)	126 (67.0)	210 (68.9)	0.38
Use of statin (%)	258 (84.6)	158 (84.0)	258 (84.6)	0.74
Use of diuretic (%)	219 (71.8)	134 (71.3)	219 (71.8)	0.80

*Proportion of surviving endothelial cells after 48 h incubation with anti-endothelial cell antibody (AECA) as described in the section “Subjects and Methods.” H/O – history of; LDL – low density lipoprotein, IQR – interquartile range, BMI – body mass index, eGFR – estimated glomerular filtration rate, BP – blood pressure, HbA_1_c – glycosylated hemoglobin.

*P*-values for all outcomes were two-sided; values <0.05 were considered statistically significant. Statistical analyses were performed using SAS, version 9.3.

## Results

### Association between endothelial cell autoantibodies and baseline risk factors

All of the 305 individuals in this analysis were men. The study population (*n* = 305) did not differ significantly in any of the mean baseline risk factor levels shown in Table 1 from all 1448 randomized VA NEPHRON D participants (data not shown). The median albuminuria level in the 305 individuals was 1501 mg/g creatinine, the mean estimated glomerular filtration rate (eGFR) was 55.2 ± 18.8 ml/min/1.73 m^2^. We observed 38% (117/305) strongly inhibitory EC activity ( ≤80% cell survival) in the immunoglobulin G (IgG) fraction of plasma. Table 1 shows the participant characteristics by AECA-mediated cell survival group. There was no significant association between AECA-mediated cell survival group with baseline kidney function or albuminuria, glycosylated hemoglobin (HbA_1_c), LDL cholesterol, systolic or diastolic blood pressure, smoking, or medication use. We observed that participants with less inhibitory AECA were younger. Individuals with strongly inhibitory AECA were more likely to have diabetic retinopathy, but less likely to have a history of a prior macrovascular event. There was a trend of a significant association between strongly inhibitory AECA and baseline atrial fibrillation history.

### Risk factors associated with primary composite renal endpoint occurrences (death, ESRD, substantial decline in eGFR)

**Figure 1:**
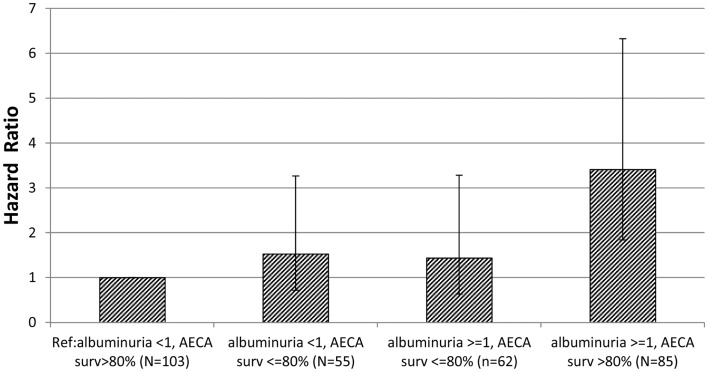
**Hazard ratio of time to primary endpoint occurrence by AECA-mediated cell survival, albuminuria subgroup is shown**. The hazard ratio (bar height) and 95% confidence intervals (brackets) were computed from Cox regression survival analysis of time to first post-randomization primary endpoint occurrence using albuminuria <1 g/g creatinine, AECA-mediated cell survival >80% as the (Ref) – reference group.

Fifty-eight primary end points were experienced by the 305 participants. In Cox regression analysis, there was a significant interaction effect of baseline AECA-mediated cell survival and baseline albuminuria level on the hazard rate (HR) for primary composite endpoint occurrence (*P* = 0.017) (Table 2). Compared to reference participants with low baseline albuminuria level ( <1 g/g creat) and lacking strongly inhibitory anti-EC antibodies ( >80% cell survival), patients with albuminuria >1 g/g and lacking strongly inhibitory anti-EC antibodies ( >80% cell survival) had a significantly higher hazard for the primary endpoint, HR, 3.41 (CI, 1.84–6.33; *P* < 0.001); whereas participants with strongly inhibitory antibodies and higher baseline albuminuria ( ≥1 g/g creatinine) or those with strongly inhibitory antibodies and lower baseline albuminuria ( <1 g/g creatinine) had a non-significant hazard for primary endpoint occurrence HR, 1.44 (CI, 0.63–3.28) and HR, 1.53 (CI, 0.72–3.27), respectively (Figure 1; Table 2).

**Table 2:** **Cox regression models of risk factors associated with primary endpoint**.

Parameter	Hazard ratio	Lower 95% CI	Upper 95% CI	P-value
Treatment (ARB + ACEI vs. ARB alone)	0.78	0.48	1.27	0.312
Albuminuria ( <1 g/g)
AECA cell survival ≤80%	1.53	0.72	3.27	0.271
AECA cell survival >80%	1			
Albuminuria ( ≥1 g/g)
AECA cell survival ≤80%	1.44	0.63	3.28	0.385
AECA cell survival >80%	3.41	1.84	6.33	<0.001
GFRLT60 ( < 60 vs. ≥60)	1.16	0.69	1.96	0.580
Age	0.97	0.94	1.00	0.078
Prior macrovascular event	0.80	0.49	1.32	0.388

*GFRLT – glomerular filtration rate <60 ml/min/1.73 m^2^*.

There was no significant association of time to first post-baseline primary end point occurrence with treatment (*P* = 0.312), baseline eGFR ( <60 vs. ≥60 mL/min/1.73 m^2^) (*P* = 0.580), prior macrovascular event (*P* = 0.388) or age (*P* = 0.078) (Table 2). There was no significant association of baseline albuminuria × eGFR, baseline albuminuria × age, baseline albuminuria × treatment, or baseline AECA-mediated cell survival × treatment to time to primary endpoint.

In Cox regression analysis that did not include albuminuria × ACEA-mediated cell survival interaction, there was a significant albuminuria effect on the primary endpoint: [HR, 2.25; 95% confidence intervals (CI), 1.37–3.69; *P* = 0.001] (not shown in Table 2).

### Association between AECA-mediated cell survival and primary endpoint by treatment group and baseline albuminuria

We next examined the association between AECA-mediated cell survival and primary endpoint occurrence in the two treatment groups stratified by baseline albuminuria level (Table 3). Among participants having baseline albuminuria level <1 g/g creatinine, a primary endpoint occurrence was experienced by 19% of ARB-treated and 17% of ACEi + ARB-treated participants. Among patients having baseline albuminuria level ≥1 g/g creatinine, a primary endpoint occurrence was experienced by 29% of ARB-treated and 25% of ACEi + ARB-treated participants (*P* = 0.08 compared to lower albuminuria subgroups) (Table 3). In the higher baseline albuminuria subgroup ( ≥1 g/g creatinine) primary endpoint occurrences were experienced by 19% of ARB-treated patients having AECA-mediated cell survival ≤80 and 38% of ARB-treated patients having AECA-mediated cell survival >80% (Table 3). Primary endpoint occurrences were experienced by 8% of ACEi + ARB-treated individuals having AECA-mediated cell survival ≤80 and 35% of ACEi + ARB-treated participants having AECA-mediated cell survival >80%, in the high albuminuria subgroup (Table 3).

**Table 3:** **Association between anti-endothelial cell antibody-mediated cell survival group and primary endpoint by treatment and baseline albuminuria group**.

	Treatment	AECA cell surv. ≤80%			AECA cell surv. >80%			Total		
		*N*	No. of prim endpoint	%	*N*	No. of prim endpoint	%	*N*	No. of prim endpoint	%
**ALBUMINURIA**										
<1 g/g creat	ARB alone	26	5	19	55	10	18	81	15	19
	ACEI + ARB	29	7	24	48	6	13	77	13	17
	Subtotal	55	12	22	103	16	16	158	28	18
≥1 g/g creat	ARB alone	36	7	19	42	16	38	78	23	29
	ACEI + ARB	26	2	8	43	15	35	69	17	25
	Subtotal	62	9	15	85	31	36	147	40	27^a^

*^a^*P* = 0.08 compared to primary (prim) endpoint occurrence (subtotal) in lower albuminuria subgroup*.

## Discussion

The present data are the first to suggest a substantially increased prevalence (38%) of strongly inhibitory plasma anti-EC autoantibodies in proteinuric adult type 2 diabetic nephropathy. These findings are consistent with prior reports of an association between strongly inhibitory EC autoantibodies and proteinuric type 2 diabetic nephropathy (12, 18). Taken together, they suggest that strongly inhibitory anti-EC autoantibodies correlate with (and may have a pathogenic role) the development of nephropathy in the older adult type 2 DM.

Several of the known properties of anti-EC antibodies suggest their possible involvement in mediating one or more pathological findings [loss of podocytes, loss of glomerular ECs, or decreased vascular endothelial cell growth factor (VEGF) expression] reported in renal biopsy specimens in human diabetic nephropathy (19). For example, the antibodies exhibited high affinity binding to heparin Sepharose columns (20), binding to heparan sulfate proteoglycan (HSPG) (18) and were significantly associated with low or undetectable plasma bFGF (20) and with increased albuminuria level in diabetic subsets having one or more microvascular complications (18). Since glomerular loss of HSPG precedes the development of diabetic albuminuria (21), anti-EC antibodies, which can target HSPG or HSPG–heparin-binding growth factor (HBGF) complexes in tissue(s) or plasma may interfere with HBGF-mediated function(s), e.g., survival-promotion in podocytes or in glomerular capillary ECs mediated by VEGF and/or bFGF, respectively (22). In addition, strongly inhibitory anti-EC antibodies induced EC apoptosis and caused strong EC contraction by activating the RhoA/Rho kinase (ROCK) signaling pathway in ECs (12). The RhoA/ROCK signaling pathway plays a key role in mediating EC barrier function (23) and its activation has been implicated in pathophysiology in animal models of proteinuric diabetic nephropathy (24). The current finding of a significant association between potent inhibitory EC autoantibodies and higher baseline prevalence of retinopathy is consistent with prior reports of an association between anti-EC inhibitory autoantibodies and coexisting diabetic microvascular complications, such as macular edema, nephropathy, and painful neuropathy (15, 18). Strongly inhibitory anti-EC, diabetic antibodies may play a role in a subset of diabetic complications, which share increased capillary permeability as an underlying mechanism.

Angiotensin II increases bFGF and transforming growth factor beta (TGF-β) expression in vascular smooth muscle cells (25); bFGF and TGF-β (acting separately or coordinately) promote several of the key cellular processes underlying glomerulosclerosis and/or tubulointerstitial fibrosis, which are thought to underlie declining renal function in diabetic nephropathy (20, 26–28). Although bFGF does not normally circulate in normal adult, non-pregnant plasma, its levels increased substantially in diabetic micro- and macroalbuminuria (29) and were significantly associated with increased plasma plasminogen activator inhibitor-1 (29), an important mediator in diabetic glomerulosclerosis (30). The present finding of a significant inverse association between strongly inhibitory anti-EC antibodies and the HR for primary composite endpoint in VA NEPHRON-D participants with higher baseline albuminuria is consistent with a role for decreased local tissue bioavailability of bFGF (in association with strongly inhibitory anti-EC antibodies) as was previously reported in subsets of older adult type 2 DM (12, 15, 18).

Less inhibitory and/or stimulatory anti-EC antibodies (which together encompass AECA-mediated cell survival >80%) may be a marker involved in mediating the increased hazard for primary endpoint in patients having higher albuminuria. Prior work in older adult type 2 DM (31) and in cancer subsets (14) demonstrated that less inhibitory anti-EC antibodies frequently coexisted with increased bFGF and with a pool of high titer (FGF-like) growth stimulatory antibodies. In a preliminary investigation in a cohort with more advanced baseline type 2 DM CKD (eGFR ranging from 15 to 25 ml/min/1.73 m^2^) than in the baseline VA-NEPHRON-D, participants were 2.5 times more likely to have had (low titer) weakly inhibitory and (high titer) moderately stimulatory (FGF-like) EC plasma antibodies (i.e., AECA >80%) than AECA ≤80%, i.e., strongly inhibitory anti-EC antibodies at both low and high titers tested [M. Zimering, unpublished observation]. Growth stimulatory antibodies having properties resembling those of bFGF stimulated proliferation in both ECs and in fibroblasts (14). They may contribute to progression in diabetic nephropathy through actions similar to those of bFGF, which stimulated fibroblast proliferation in the renal interstitium (28).

The albuminuria × anti-EC antibody-mediated cell survival interaction demonstrated here suggests that higher albuminuria is necessary, but may not be sufficient to explain a substantial degree of the variability in renal composite endpoint occurrence in an older, high-risk diabetic nephropathy population. Endothelial damage and inflammation have been identified as possible markers of residual high risk in subsets of diabetic nephropathy (10). Basic FGF is released as a result of endothelial damage (32) and tubulointerstitial inflammation is thought to be mediated in part by increased albuminuria and by RAS activation (22). Taken together, bFGF released ectopically as a result of endothelial damage and pro-inflammatory cytokines upregulated (in part) as a result of hyperglycemia and/or obesity in type 2 diabetes may facilitate (through as yet poorly-defined mechanisms) immune interactions favoring a transition from strongly inhibitory (AECA ≤80%) to less inhibitory or stimulatory AECA having cell-mediated survival >80% in subsets of advancing diabetic CKD. Increased expression of high affinity HSPG binding sites for bFGF reported in the renal interstitium during tubulointerstitial fibrosis (33) may provide an additional mechanism for strongly inhibitory or FGF-like stimulatory autoantibodies to differentially modulate the rate of tubulointerstitial fibrosis thereby slowing or accelerating, respectively, the rate of decline in renal function in diabetic nephropathy. Increased glomerular capillary permeability underlies higher albuminuria level suggesting that higher albuminuria level facilitates access to the renal interstitium of IgG present at high titer, i.e., strongly inhibitory (in AECA ≤80%) or stimulatory IgG (in AECA >80%), respectively. In this way, local cell proliferation in the renal interstitium is likely to be modulated by EC autoantibodies occurring near the extremes along a spectrum from strongly inhibitory to stimulatory EC activity.

Among several limitations of our study, one is that the findings may only reflect the experience of middle-aged or older men with type 2 DM. More study is needed in additional and larger diabetic populations to confirm a role for AECA-mediated cell survival/albuminuria interaction effect on primary endpoint occurrence. Non-diabetic renal disease may have coexisted in a small subset of adult type 2 diabetes and possibly contributed to the observed effect of AECA-mediated cell survival/albuminuria interaction on the overall rate of primary end point occurrence. An increased prevalence of anti-EC antibodies was reported in advanced non-diabetic CKD compared to a normal population (34) and similar kinds of effects in ECs (e.g., increased cytosolic calcium and actin stress fiber formation) were observed in autoantibodies from non-diabetic (34) or diabetic CKD autoantibodies (12). Still, we could not determine whether our findings may be generalizable to non-diabetic CKD populations. More detailed characterization of the diabetic nephropathy IgG autoantibodies (e.g., its subclass specificity) will also require further study. In our preliminary experiments, strongly inhibitory anti-EC activity was stable after storage (at 0–4°C) for at least 9 months, and addition of 10 μg/mL concentrations in three of three strongly inhibitory anti-EC antibodies tested to EC cells caused rapid EC contraction (within 1 min) and stress fiber formation. After fourfold dilution (in a subset of the protein A eluates) from a starting 30 μg/mL concentration, mean activity in protein A eluates in strongly inhibitory anti-EC antibodies still significantly exceeded mean activity in protein A eluates from patients having less inhibitory anti-EC antibodies (79.7 ± 4.9% vs. 97.3 + 3.4%; *p* < 0.001, *n* = 9 patients). The latter observation is consistent with a possible role for higher titer, strongly inhibitory anti-EC antibodies in modulating HBGF bioavailability in the renal interstitium.

In summary, the current findings demonstrate involvement of a novel, putative pathogenic factor, i.e., anti-EC autoantibodies, as a significant modulator of the occurrence of the composite endpoint of renal function decline, ESRD, or death in adult proteinuric, type 2 DM. Much more study is needed to determine whether the albuminuria/AECA interaction implies a dual role for strongly inhibitory and/or stimulatory antibodies associated with less inhibitory antibodies in providing a brake for or accelerating growth factor-driven cellular processes underlying declining renal function (or premature death) in adult type 2 DM nephropathy.

## Conflict of Interest Statement

Dr. Nicholas Emanuele received honoraria as member of the Speaker’s bureau for Merck and Co., Inc. The other co-authors declare that the research was conducted in the absence of any commercial or financial relationships that could be construed as a potential conflict of interest.
